# Acquired Factor VIII Inhibitor Presenting as Occult GI Bleeding

**DOI:** 10.1155/2018/1910854

**Published:** 2018-06-19

**Authors:** Carlos I. Pacheco Cano, Marilyn A. Arosemena, Roumen B. Iordanov, Ankitha Lingamaneni, Ankur Aneja

**Affiliations:** ^1^Department of Medicine, Jackson Memorial Hospital, University of Miami, Miami, FL, USA; ^2^University of Miami Miller School of Medicine, Miami, FL, USA

## Abstract

An acquired coagulation factor deficiency is characterized by acquired autoantibodies against specific clotting factors in those without diagnosed hemophilia. It is a relatively rare condition with an incidence of approximately one case per million per year. We present a case report of an elderly male who initially presented with an occult GI bleed that was identified with a positive fecal occult blood test result. This is the first case reported to our knowledge of an acquired factor inhibitor deficiency presenting in this manner. We postulate the importance of acquired factor inhibitors in the setting of unexplained anemia given absence of overt clinical symptoms that could contribute to aggravate an established GI bleed, especially in the elderly population.

## 1. Introduction

An acquired coagulation factor deficiency is characterized by acquired autoantibodies against specific clotting factors in non-hemophiliacs. It is a relatively rare condition with an incidence of approximately one case per million per year. Autoantibodies can develop against any coagulation cascade factor, but most commonly to VIII, IX, and XI. While congenital forms of hemophilia are characterized by an early age of onset, the median age of presentation for the acquired deficiency is between 60 and 67 years. Clinical presentation also differs significantly; congenital hemophilia presents primarily with hemarthrosis in contrast to acquired hemophilia, which presents with purpura or soft tissue bleeding [1–3].

## 2. Case Presentation

A 79-year-old man with a past medical history significant for hypertension, chronic kidney disease stage IV, dementia, and chronic obstructive pulmonary disease presented to the Emergency Department after being transferred from a correctional facility due to low hemoglobin found on routine labs. On arrival, his vital signs included a temperature of 36.5°C, heart rate of 88 bpm, respiratory rate of 22/min, blood pressure of 159/70 mmHg, and saturation of 99% on room air. Physical examination was notable for dry oral mucosa and poor dentition, a 2/6 systolic murmur best heard at the left sternal border, and hematomas on both posterior shoulders, bilateral upper arms, and the right medial forearm. The only medication the patient was taking at the time was amlodipine 10 mg daily for hypertension. Upon admission, the patient was agitated and hostile to interview and was thus treated with Haldol 5 mg IM x1. Laboratory studies were performed and showed a hemoglobin level of 6.9 g/dL (decreased from his baseline: 10 g/dL), elevated BUN at 99 mmol/L, and creatinine of 3.23 mg/dL (increased from his baseline creatinine: 2.7 mg/dL).

**Figure 1:**
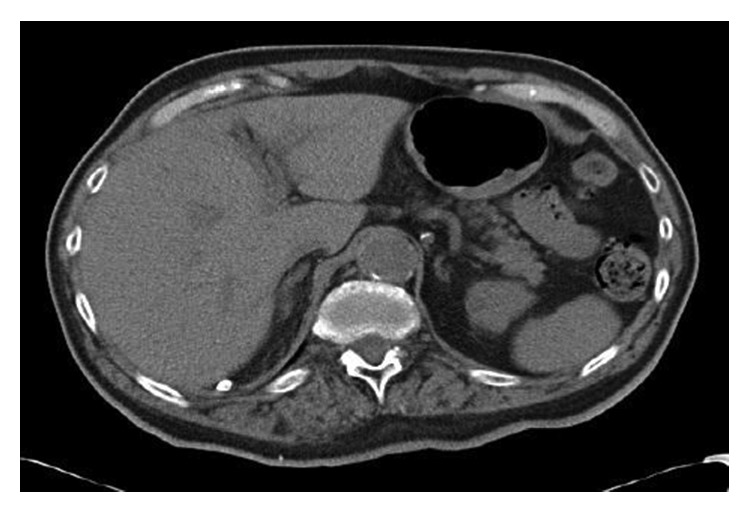
Right perinephric and right paracolic gutter fat stranding surrounding the right iliopsoas muscle.

He denied any symptoms associated with anemia such as lightheadedness, dizziness, shortness of breath, hemoptysis, or hematemesis. He stated that he was unsure if there was melena because he does not routinely inspect his stool. He was initially started on intravenous fluids and given red blood cell transfusion. Immediately after transfusion, his hemoglobin increased to 7.5 g/dL; however, hemoglobin levels continued dropping on subsequent days with the lowest level at 4.6 g/dL. As a result, the patient required a total of 6 packed red blood cell transfusions. After continued intravenous hydration and transfusions, BUN and creatinine decreased to 57 mmol/L and 2.3 mg/dL, respectively. Occult gastrointestinal (GI) bleeding was suspected due to consistent downtrending hemoglobin refractory to blood transfusions. The patient became more cooperative on subsequent days and complained of lower left quadrant abdominal pain as well as chronic bilateral leg pain. The fecal occult blood test was performed and was positive both times. CT abdomen (Figure 1) was performed to rule out intra-abdominal causes of acute anemia and showed right perinephric and right paracolic gutter fat stranding surrounding the right iliopsoas muscle which was asymmetrically enlarged without evidence of definite hyperdense intramuscular hematoma, but possible intramuscular hemorrhage or myositis.

**Figure 2:**
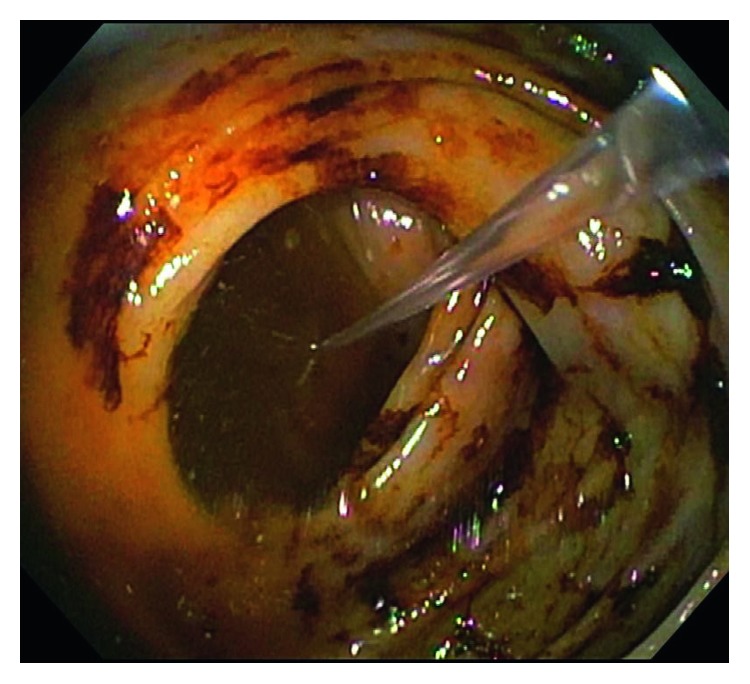
Terminal ileum containing hematin (altered blood/coffee-ground-like material) without a clear source of bleeding.

The gastroenterology team was consulted, and esophagogastroduodenoscopy (EGD) and colonoscopy were performed. EGD did not yield any abnormal results, but colonoscopy revealed terminal ileum containing hematin (altered blood/coffee-ground-like material) without a clear source of bleeding (Figure 2). Coagulation studies were subsequently performed which showed an elevated activated partial thromboplastin time (aPTT) at 101.5 seconds. Hematology service was consulted and a mixing study was ordered, which showed only partial correction of the aPTT, suggesting the possibility of a coagulation factor-specific antibody. Levels of factors VIII, IX, XI, and XII were ordered and showed factor VIII activity of <0.01 unit/mL, factor IX activity of 0.80 unit/mL, factor XI activity of 0.40 unit/mL, and factor XII activity of 0.34 unit/mL. Subsequently, the Bethesda assay was performed to measure autoantibody levels against any of these factors and showed an elevated factor VIII inhibitor level at 390 U/mL, which confirmed the diagnosis of acquired hemophilia A. The patient was started on cyclophosphamide 50 mg QD and dexamethasone 20 mg QD to inhibit the body's immune response against factor VIII. Inhibitor levels decreased to 280 U/mL after 10 days of therapy. Etiology of the patient's acquired factor VIII inhibitor was unclear, so tumor markers (PSA, CEA, CA 19-9) and CT scans (thorax and abdominopelvic) were ordered in order to rule out the underlying malignancy. All markers and imaging were within normal limits. Hemoglobin remained stable at 8 g/dL after starting appropriate treatment. Because throughout the next few weeks, the inhibitor level did not decrease greatly in response to cyclophosphamide therapy, the patient was started on weekly infusions of rituximab. The most recent factor VIII inhibitor level was down to 70.4 U/mL with stable hemoglobin at 13.2 g/dL. Hematology continues to closely monitor the quantity of the inhibitor levels and his response to treatment. In addition, the patient is advised to avoid any activity in which he may sustain trauma or fall due to his increased risk of bleeding.

## 3. Discussion

This report discusses a 79-year-old man with a newly diagnosed acquired factor VIII inhibitor initially presenting as occult gastrointestinal and intramuscular bleeds. Acquired hemophilia is a life-threatening autoimmune condition in which there is spontaneous development of antibodies against factor VIII. It is seen in 1.5 patients per million annually and mortality rates have been estimated as 7.9–22%, with most hemorrhagic deaths occurring within the initial weeks after presentation. This condition should be suspected in any patient with no personal or family history of bleeding who presents with a sudden onset bleed with no instigating factor or excessive bleeding after mild trauma [1, 4].

While there have been other studies highlighting the various presentations of acquired factor inhibitors, no other documented case has been presented with anemia in the setting of occult gastrointestinal and intramuscular bleeding. In other acquired factor VIII inhibitor cases in the literature, patients first presented with gross hematuria or acute evident gastrointestinal bleeding [5]. Other cases highlight acquired factor VIII inhibitor in patients with underlying malignancy, interstitial lung disease, SLE, and bullous pemphigoid, and yet others presented with delayed wound healing. In this case, malignancy was ruled out and the cause of acquired factor VIII inhibitors remains uncertain [6–9].

Unlike most patients with this condition who initially present with soft tissue bleeds, our patient presented with an occult GI bleed that was identified with positive fecal occult blood testing. Further workup with colonoscopy and EGD showed no specific source of bleeding. Coagulation studies revealed an elevated aPTT level, and a mixing study showed only partial correction, which raised suspicion for an acquired coagulation factor inhibitor. Further testing with the Bethesda assay showed elevated levels of factor VIII inhibitor and confirmed the diagnosis. This is an unusual presentation of the disease, and there have not been any documented cases of acquired hemophilia presenting with anemia in the setting of an occult GI bleed. This case report prompts clinicians to consider acquired factor inhibitors in the setting of occult bleeding and shows that such a presentation warrants further workup, including mixing study and Bethesda assay, when coagulation results are abnormal. Furthermore, when an elderly patient presents in this manner, secondary causes of acquired hemophilia such as malignancy must be worked up with age-appropriate cancer screening. We believe in this case, the Hb level was stabilized after the therapy was initiated and the level of bleeding noticed through the gastrointestinal tract was not severe enough to cause a drop on it in the short duration of hospitalization that the patient experienced on this occasion.

## 4. Conclusion

Acquired hemophilia is a rare condition that presents in approximately one in one million people and often has fatal consequences due to its late diagnosis and lack of awareness amongst practicing physicians. Although it most often presents with overt soft tissue bleeding, it is important for clinicians to suspect this abnormality in patients with atypical presentations, such as occult GI bleeding. Mixing studies can be used to differentiate coagulation factor deficiency and the presence of a specific factor inhibitor, failure to correct prompts further workup with the Bethesda assay to rule out acquired hemophilia. Though more than half of the cases arise idiopathically, 10% of the cases are associated with malignancy, and cancer work-up is warranted in such patients.
